# Case Report: Hormone receptor-positive uterine mesonephric-like adenocarcinoma

**DOI:** 10.3389/fmed.2026.1787557

**Published:** 2026-04-20

**Authors:** Ting Luo, Jianing Shen, Yongjun Zeng, Kang Xie, Nie Xu

**Affiliations:** 1 Department of Oncology, The Research Institute of Integrated TCM & Western Medicine of Chengdu University of Chinese Medicine, Chengdu Integrated TCM and Western Medicine Hospital, Chengdu, China; 2 School of Basic Medical Sciences, Chengdu University of Traditional Chinese Medicine, Chengdu, China; 3 Department of Pathology, Chengdu Integrated TCM and Western Medicine Hospital, Chengdu, China

**Keywords:** case report, estrogen receptor, mesonephric-like adenocarcinoma, progesterone receptor, uterus

## Abstract

**Background:**

Uterine mesonephric-like adenocarcinoma (MLA) is a rare and biologically aggressive subtype of endometrial carcinoma, estrogen receptor (ER) and progesterone receptor (PR) are typically absent or exhibit very low expression in this type of cancer. The cornerstone of treatment is still surgical resection, frequently in combination with chemotherapy or radiation therapy. There is currently disagreement over the optimal therapeutic regimen.

**Case presentation:**

A 60-year-old postmenopausal woman presented with vaginal bleeding as the initial symptom. Computed tomography (CT) imaging revealed a uterine mass, and postoperative histopathology confirmed a diagnosis of mesonephric-like adenocarcinoma of the uterus. This case demonstrates dual ER/PR positivity, representing the first reported instance of this rare clinical subtype.

**Conclusion:**

This study aims to enhance clinicians’ recognition and management of uterine mesonephric-like adenocarcinoma. MLA with hormone receptor expression may exhibit distinct biological behavior. Future studies with larger cohorts and longer follow-up are needed to explore whether these tumors could represent a candidate for endocrine-based therapeutic approaches.

## Introduction

Uterine mesonephric-like adenocarcinoma (MLA) is an exceedingly rare and aggressive malignancy of the female reproductive tract, accounting for less than 1% of all gynecologic cancers. It is frequently misdiagnosed due to histopathologic overlap with other gynecologic neoplasms. Clinical presentations are typically non-specific, with abnormal vaginal bleeding or postmenopausal bleeding being the most common initial symptoms. Some patients may remain asymptomatic during early stages, with incidental detection on imaging studies (1). This case report describes a patient with uterine mesonephric-like adenocarcinoma presenting with vaginal bleeding as the initial symptom. The tumor exhibited high expression of ER and PR. The unique histological features, immunohistochemical expression, prognosis and treatment of the disease can enhance clinicians’ recognition, thereby assisting in clinical differentiation and diagnosis.

## Case presentation

A 60-year-old postmenopausal woman presented with intermittent, scant vaginal bleeding and low back pain that had persisted for 1 month without seeking timely medical attention. Her medical history was significant only for cataract surgery, and there was no family history of malignancy. A gynecological examination revealed normal vaginal mucosa with minimal discharge, an atrophic and smooth cervix without evidence of endocervical bleeding, and an anteverted, soft uterus. A palpable mass was noted in the right adnexal region. The initial differential diagnoses included endometrial hyperplasia and a right adnexal tumor.

**FIGURE 1:**
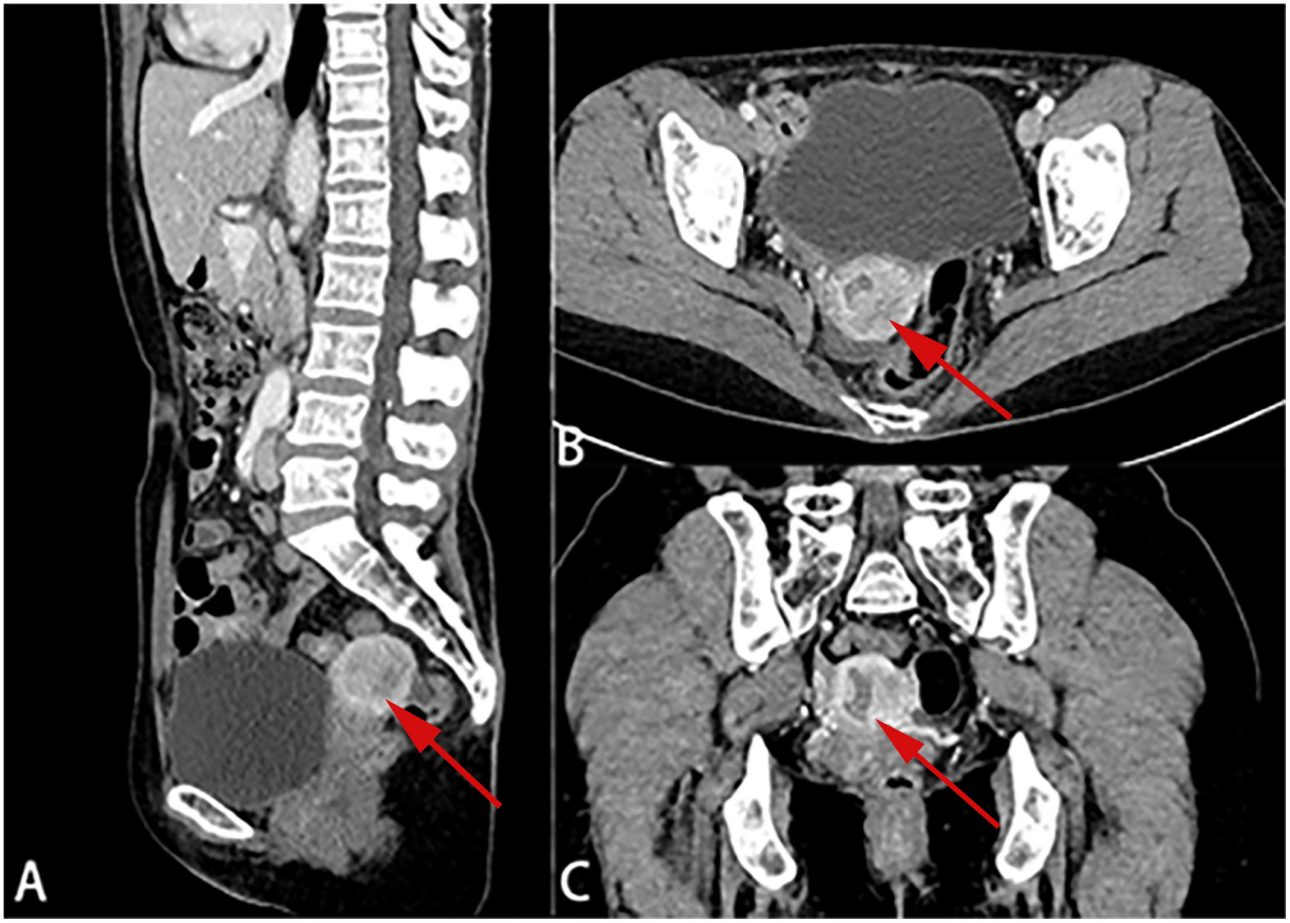
**(A–C)** Preoperative pelvic contrast-enhanced computed tomography (CT) demonstrated irregular thickening of the endometrium with heterogeneous enhancement.

Transvaginal ultrasound demonstrated endometrial thickening of approximately 1.2 cm with heterogeneous echogenicity and abundant vascular signals. In the right adnexal region, a cystic mass measuring 3.2 × 4.2 cm with well-defined borders and a regular shape was observed. Contrast-enhanced pelvic CT revealed heterogeneous endometrial thickening with notable contrast uptake (Figure 1), while the right adnexal hypodensity exhibited no enhancement. Hematologic tests indicated mild leukopenia and thrombocytopenia, Human epididymis protein 4 (HE4) was increased at 190 pmol/L, whereas CA125 and CEA were normal. Chest and abdominal CT scans, as well as cardiac echocardiography, demonstrated no significant abnormalities. Pathological examination of the diagnostic curettage specimens confirmed the presence of adenocarcinoma, thereby supporting a preliminary diagnosis of endometrial adenocarcinoma (Histological grade G3) with a concurrent ovarian cyst.

The patient underwent single-port laparoscopic hysterectomy with bilateral adnexal resection. Following hysterectomy, a cauliflower-like mass measuring approximately 3 × 2.5 × 2 cm was identified on the posterior uterine wall. The lesion infiltrated the myometrium but did not extend beyond the mid-myometrial layer. The operation lasted 4 h, and a drainage tube was placed postoperatively. Hemorrhagic fluid persisted for 3 days but resolved with hemostatic and antimicrobial therapy.

**FIGURE 2:**
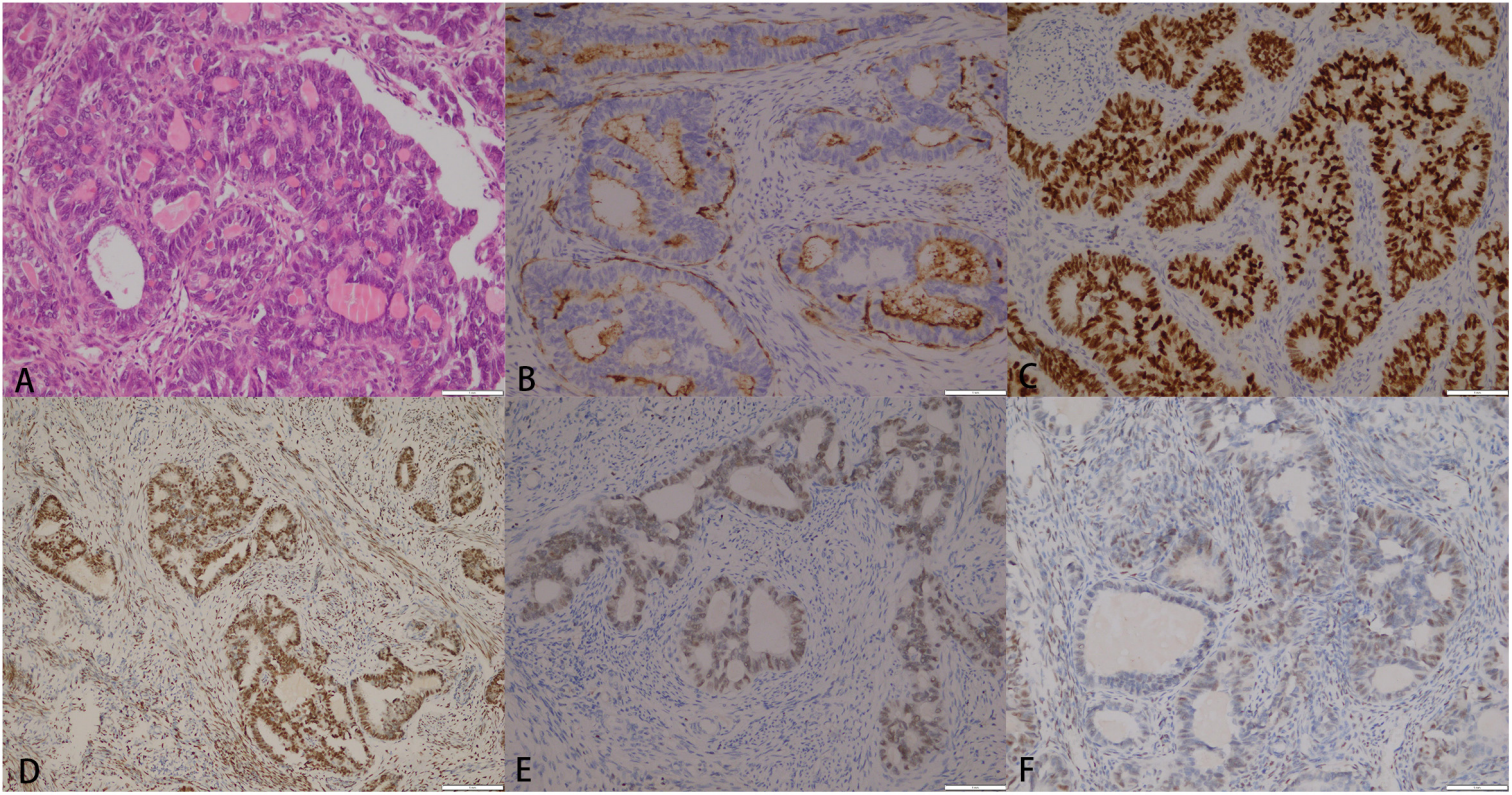
**(A)** (HE × 200) Histopathological examination revealed an infiltrative growth pattern of adenoid and sieve-like adenocarcinoma within the myometrium. The neoplastic glands exhibited characteristic eosinophilic luminal secretions. Tumor cells were arranged in pseudocomplex tubular structures and composed of cuboidal to columnar cells with eosinophilic cytoplasm, indistinct cell borders, nuclear crowding and overlapping, small nucleoli, and nuclear grooves. **(B)** (IHC × 200) CD10 was positive for luminal margins. **(C)** (IHC × 200) TTF-1 exhibited diffuse strong positie expression in the nucleus. **(D)** (IHC × 200) estrogen receptor (ER) is moderate to strong positive in 60% tumor cells. **(E)** (IHC × 200) GATA 3 exhibited positive expression in the nucleus. **(F)** (IHC × 200) progesterone receptor (PR) is moderate to weak positive in 30% tumor cells.

During the surgery, lymph node dissection was performed, and the pathology showed no metastasis in the left pelvic lymph nodes (0/5), right pelvic lymph nodes (0/8), and para-aortic lymph nodes (0/3). Histopathologic examination demonstrated an adenoid and squamous adenocarcinoma with infiltrative growth into the uterine myometrium. The lumen contained characteristic eosinophilic secretions, consistent with MLA (Figure 2A). Tumor cells displayed pseudostratified cuboidal to columnar morphology, with eosinophilic cytoplasm, indistinct borders, crowded overlapping nuclei, small nucleoli, and nuclear grooves.

A semi-quantitative scoring system was used to identify the percentage of tumor cells showing positive staining. ER and PR positive were defined as >1% of tumor cell nuclei staining positively with any intensity. Immunohistochemistry demonstrated diffuse and strong nuclear expression of TTF-1, cytoplasmic expression of CD10, and nuclear positivity for GATA3 (Figures 2B–D). Diffuse ER positivity ranges from mild to robust in about 60% of tumor cells, while PR showed moderate to weak positivity in about 30% (Figures 2E, F). Additional findings included nuclear Pax-8 positivity, preserved mismatch repair (MMR) protein expression (MLH1, PMS2, MSH2, MSH6), retained PTEN, and block-type positivity for P16. Negative immunoreactivity for calretinin, inhibin-α, and WT1 effectively excluded sex cord-stromal and serous carcinoma subtypes. The Ki-67 proliferation index was approximately 40%.

The final diagnosis was mesonephric-like adenocarcinoma of the uterus, FIGO stage IB, ER(+), PR(+). Postoperatively, serum HE4 decreased to 51.7 pmol/L. The patient was discharged after 2 weeks. Given the high risk of distant metastasis and recurrence associated with MLA, adjuvant chemotherapy or endocrine therapy was recommended; however, the patient declined further treatment. At the 1-year follow-up, pelvic MRI revealed no evidence of recurrence.

I was somewhat concerned when I initially learned of the diagnosis. But my doctors took the time to make sure I understood everything. They addressed all of my inquiries and gave me more confidence over the course of treatment. I’ve finished the surgery, and even though there were a few challenging days, the medical staff helped me every step of the way. Now that I’m feeling better, I’m happy to be returning to my regular activities. I hope that sharing my story can help other patients facing similar diagnoses and contribute to medical knowledge about this rare condition.

## Discussion

Reports of mesonephric-like adenocarcinoma (MLA) of the uterus are rare, accounting for only 0.7%–3% of all uterine body cancer (2). Endometrial biopsy is prone to misclassification and is difficult to get an accurate pathology diagnosis. The study describes a rare instance of uterine mesonephric-like adenocarcinoma with elevated ER and PR expression, which has particular clinical reference value.

Mesonephric-like adenocarcinoma arises from Müllerian duct–derived structures that differentiate along the mesonephric pathway, rather than from true mesonephric remnants, and is frequently associated with endometriosis (1). Compared with other uterine malignancies, MLA is characterized by deep myometrial invasion, larger tumor size, a higher risk of lymphovascular invasion and distant metastasis, and increased recurrence rates, which collectively contribute to poor prognosis. Reported cases span a wide age range but occur most commonly in middle-aged women. In this case, the patient is an elderly female presenting with a relatively small tumor size and shallow invasion depth. The tumor has not yet invaded surrounding tissues or organs, which further complicates early detection.

Mesonephric-like adenocarcinoma exhibits characteristic histological features, including a heterogeneous mixture of growth patterns such as tubular, glandular, papillary, solid/diffuse, reticular, and glomeruloid (2, 3). Nuclear morphology typically demonstrates mild to moderate cytological atypia, with chromatin ranging from hyalinized to vacuolated and occasionally showing overlap, keratinization, or grooving. Hyperchromatic nuclei may also be observed, while nucleoli are generally small or inconspicuous. A hallmark finding is the presence of tightly clustered tubular structures containing eosinophilic, glassy secretions (4, 5).

Beyond morphology, diagnosis of MLA requires supportive immunophenotyping. GATA3 (GATA-binding protein 3), TTF-1 (thyroid transcription factor-1), and CD10 are typically positive in most MLA cases and represent key immunohistochemical markers (5, 6). Notably, TTF-1 and GATA3 may display reciprocal staining patterns, with one marker positive while the other is negative (5). GATA3 is regarded as a sensitive and specific marker for mesonephric lesions, with reported sensitivity and specificity approaching 98% in distinguishing these lesions from cervical and endometrial cancers (7). GATA3 staining is usually weak to moderate in intensity and involves a low proportion of tumor cells (<10%) (5), whereas TTF-1 and CD10 more commonly demonstrate moderate-intensity diffuse positivity.

Mesonephric-like adenocarcinoma shows morphologic and immunophenotypic similarity to MA (mesonephric adenocarcinomas) but originates from the Müllerian duct system, which gives rise to the uterus, fallopian tubes, and endometrium. MLA primarily occurs in the endometrium and ovaries, representing its most critical distinction from true mesonephric adenocarcinoma. Histologically and immunohistochemically, MLA is nearly indistinguishable from MA; however, TTF-1 expression is observed in approximately 30%–40% of MLA cases, whereas MA is typically TTF-1-negative, providing an important differential diagnostic clue (8). The characteristics of Mesonephric-like adenocarcinoma, mesonephric adenocarcinoma, and endometrial carcinoma are compared in the Table 1.

**TABLE 1:** Comparison of clinicopathological and molecular characteristics among mesonephric-like adenocarcinoma, mesonephric adenocarcinoma, and endometrial carcinoma.

Feature	MLA	MA	EC
Incidence	Rare	Extremely rare	Common
Median age	60 years	Varies widely	64 years
Primary site	Uterine corpus and ovary	Uterine cervix	Endometrium
Histogenesis	Müllerian	Mesonephric (Wolffian) duct remnants	Müllerian
Cellular morphology	Multiple growth patterns	Multiple growth patterns	Monotypic growth pattern
Nuclear features	Moderate atypia, chromatin appears clear to vesicular with small nucleoli, nuclear bending and hyperchromasia	Mild to moderate atypia, unevenly distributed, coarsely granular chromatin, nuclear hyperchromasia less pronounced than in MLA	Mild to moderate atypia, enlarged, oval to round nuclei, vesicular chromatin, variable eosinophilic nucleoli, mitotic activity may be brisk
Metaplasia	Squamous, mucinous, or ciliated metaplasia typically absent	Metaplasia absent	Squamous metaplasia
Immunohistochemistry	GATA3+/TTF1+, ER/PR−, PAX8+ (diffuse), CD10+ (luminal margin), PAX2+, SOX17−	GATA3+, TTF1− or focal+, ER/PR−, PAX8+, CD10+, p16+ (focal)	ER/PR+, p53 wild-type, PTEN (70%–80%loss)
TCGA classification	NSMP subgroup	Not TCGA classification, but genomic features are highly similar to MLA	POLE (ultramutated), MSI-H (hypermutated), copy-number low/NSMP, copy-number high/p53 mutant

MA, mesonephric adenocarcinoma; EC, endometrial carcinoma; NSMP, no specific molecular profile; TCGA, The Cancer Genome Atlas.

Microscopically, this case demonstrated infiltration growth of malignant glands within the uterine myometrium, featuring a mixed architectural pattern of glandular, tubular, and reticular or mesenchymal structures. The glandular lumina contains eosinophilic secretions characteristic of mesonephric-like adenocarsionama (MLA). Immunohistochemical analysis shows strong PAX8 positivity, with negative staining for calretinin (CR) and WT-1, consistent with a Müllerian origin. Patchy p16 positivity suggested a non-HPV-related adenocarcinoma (9).

In the differential diagnosis, uterine MLA must be distinguished from endometrioid adenocarcinoma and uterine collision tumors. The key distinguishing features include TTF-1 expression, spatial distribution, architectural morphology, and immunophenotypic profile. Positive TTF-1 expression serves as a critical diagnostic indicator favoring MLA, and approximately 8% of TTF-1–positive endometrioid adenocarcinomas may in fact represent misclassified MLAs (10).

Collision tumors are defined as two or more histologically distinct neoplasms coexisting within the same organ or anatomical site, separated by normal stroma and lacking any histological transition or blending zones. This definition fundamentally distinguishes them from “mixed tumors,” in which components show morphological intermingling. Collision tumors have been described in multiple organs, including the skin, thyroid, lung, gastrointestinal tract, and urogenital system; however, their occurrence in the uterine body or ovaries remains exceedingly rare.

We further investigated previous case reports and literature reviews on uterine collision tumors and identified 14 cases of uterine collision tumors (11–14). The reported age at diagnosis ranged from 36 to 85 years, predominantly affecting postmenopausal women. Clinical presentations were non-specific, most commonly manifesting as postmenopausal vaginal bleeding. Preoperative misdiagnosis was common, as the tumors were often mistaken for a single histologic entity. Definitive diagnosis requires postoperative histopathological and immunohistochemical examination. Histologic combinations were diverse, with carcinoma–sarcoma coexistence being the most frequent pattern. One report described a rare triple collision tumor comprising a Müllerian mixed tumor, serous carcinoma, and endometrioid adenocarcinoma. Surgical resection remains the mainstay of treatment, with adjuvant therapy tailored to the most aggressive component and highest stage. Prognosis varies considerably and is primarily determined by the most malignant element, generally resulting in poorer outcomes than those observed in monotypic tumors. In the present case, only adenocarcinoma components were observed in the endometrium without evidence of other histologic types. Both TTF-1 and GATA3 exhibited diffuse nuclear positivity without focal loss or sharp demarcation, supporting a diagnosis of uterine mesonephric-like adenocarcinoma rather than a collision tumor.

On the other hand, this patient demonstrated a rare dual-positive expression profile, with high ER expression (60%) and low-to-moderate PR expression (30%). Although such findings have not been previously documented in the literature, the tumor’s morphology and immunophenotype conform to established diagnostic criteria, suggesting biological behavior distinct from classic MLA. Prior studies have reported that MLA typically shows absent or minimal hormone receptor expression, with ER and PR positivity rates generally ≤10% (15, 16). Moreover, ER/PR negativity has been identified as an independent risk factor for recurrence and mortality in endometrial adenocarcinoma, significantly correlating with deep myometrial invasion, lymph node metastasis, and advanced disease stage. Given the aggressive nature of MLA, we recommend intensive postoperative adjuvant therapy even in early-stage disease. Based on the ER/PR-positive immunophenotype, these patients may represent a special molecular subgroup with relatively good prognosis within uterine mesonephric-like adenocarcinoma, providing potential stratification basis for subsequent prospective studies.

In this case, next-generation sequencing identified a KRAS p.G12D mutation, with no PIK3CA mutation detected. Based on the available molecular data, this case is most consistent with the NSMP subgroup of endometrial cancers. The “no molecular specific profile” (NSMP) subgroup of endometrial carcinoma and is characterized by pronounced molecular and histopathological heterogeneity (17). Approximately 30% of uterine and ovarian MLA harbor PIK3CA mutations (18), with some cases exhibiting alterations in PTEN or CTNNB1. The PIK3CA mutation is not exclusive to MLA, as it is also frequently observed in endometrial cancer. However, it was identified in a tumor exhibiting MLA morphology and an immunophenotype characterized by GATA3+ and TTF-1+. This finding provides molecular-level supporting evidence for diagnosis, complementing KRAS mutations and thereby enhancing the comprehensiveness of the diagnosis (19).

Histologically, the presence of eosinophilic or hyaline luminal secretions serves as a distinctive diagnostic feature of MLA (5). Immunohistochemistry functions as a critical adjunctive tool, while molecular testing provides further diagnostic confirmation. In this patient, the integration of the tumor’s anatomic origin, histomorphology, and immunophenotype facilitated the diagnosis of endometrial MLA.

Mesonephric-like adenocarcinoma represents a rare tumor type with highly aggressive biological behavior, most commonly metastasizing to the lungs (20). At present, there is no universally accepted standard treatment protocol. However, surgery combined with postoperative chemoradiotherapy remains the mainstay of management. The recommended surgical approach typically includes total hysterectomy with bilateral adnexectomy and pelvic lymph node dissection, with or without para-aortic lymph node dissection. The prognosis is generally poor, characterized by inferior progression-free survival (PFS) and overall survival (OS). For the first 2 years following surgery, postoperative follow-up should be strengthened. This includes scheduling chest CT and contrast-enhanced abdominopelvic CT scans for re-examination, as well as performing thorough medical history inquiries, physical examinations, and gynecological examinations every 3 months. Follow-up exams should be performed every 6 months from the third to the fifth year following surgery; after that, yearly exams are necessary.

Notably, disease stage may not reliably correlate with clinical outcomes, and aggressive adjuvant therapy is often advised even for patients with early-stage disease. Postoperative management may include adjuvant chemoradiotherapy, endocrine therapy, or other treatment modalities (21). The effectiveness of chemoradiotherapy, as well as the potential benefit of targeted therapies, requires further validation in large-scale clinical studies (22). For patients with hormone receptor positivity, a lower mortality risk has been observed compared with receptor-negative cases, suggesting the possibility of a distinct endometrial carcinoma subtype and highlighting the need to explore novel endocrine-based therapeutic strategies.

## Limitation

(1) Preoperative pelvic MRI should be performed to facilitate accurate determination of the surgical resection range. (2) MLA carries a high risk of recurrence. The follow-up period for this case was short (1 year), and further long-term follow-up is still required.

## Conclusion

Mesonephric-like adenocarcinoma is a rare malignancy characterized by insidious onset and high aggressiveness. Adjuvant therapy is generally recommended even in early-stage disease. This study reports a rare case of an ER/PR double-positive MLA patient. We speculate that the subset of MLA with hormone receptor expression may exhibit distinct biological behaviors. Future research should focus on larger multi-institutional cohorts to elucidate the prognostic significance of hormone receptor expression in MLA, while exploring the potential role of endocrine therapy.

## Data Availability

The original contributions presented in this study are included in this article/supplementary materials, further inquiries can be directed to the corresponding author.
